# The efficacy of elevating anandamide via inhibition of fatty acid amide hydrolase (FAAH) combined with internet-delivered cognitive behavioral therapy in the treatment of post-traumatic stress disorder: a randomized, placebo-controlled clinical trial

**DOI:** 10.1038/s41386-025-02128-w

**Published:** 2025-05-17

**Authors:** Leah M. Mayo, Emelie Gauffin, Gavin N. Petrie, Ryann Tansey, Raegan Mazurka, Connor J. Haggarty, Madeleine R. Jones, Hilda Engelbrektsson, Victoria Aminoff, Anisja Hühne-Landgraf, Mark E. Schmidt, Darrel J. Pemberton, Cecilia Fredlund, Lars Östman, Hanna Karlsson, Andreas Löfberg, Michal Pietrzak, Gerhard Andersson, Andrea Johansson Capusan, Matthew N. Hill, Markus Heilig

**Affiliations:** 1 Department of Psychiatry, Mathison Centre for Mental Health Research and Education, Hotchkiss Brain Institute, University of Calgary, Calgary, AB Canada; 2 Center for Social and Affective Neuroscience, Department of Biomedical and Clinical Sciences, Linköping University, Linköping, Sweden; 3 Department of Psychiatry, Linköping University Hospital, Linköping, Sweden; 4 Circadian Biology Group, Section of Molecular Neurobiology, Department of Psychiatry and Psychotherapy, Ludwig Maximilian University, Munich, Germany; 5 Janssen Pharmaceutica, NV Beerse, Belgium; 6 Barnafrid, Swedish National Center on Violence Against Children, Linköping University, Linköping, Sweden; 7 Department of Behavioral Sciences and Learning, Linköping University, Linköping, Sweden

**Keywords:** Post-traumatic stress disorder, Drug development

## Abstract

Post-traumatic stress disorder (PTSD) is a severe mental health disorder with limited treatment options. Gold standard treatment includes cognitive behavioral therapies (CBT) that incorporate exposure to traumatic memories to facilitate extinction. CBT can be effective in PTSD, but effects are incomplete and symptoms are prone to spontaneous return. Pharmacologically facilitating fear extinction could potentiate the effects of exposure-based therapy. Here, we explored whether targeting the endocannabinoid (eCB) system, a neuromodulatory system critically involved in fear extinction, would promote the efficacy of exposure-based CBT. Specifically, we tested the effects of elevating the eCB ligand anandamide (AEA) via inhibition of its main degradative enzyme, fatty acid amide hydrolase (FAAH). In this double-blind, placebo-controlled study, patients with PTSD (*N* = 100; 85 women) were randomized to the FAAH inhibitor (FAAHi) JNJ-42165279 (25 mg b.i.d.) or placebo for 12 weeks. In weeks 5–12, all participants completed an internet-delivered CBT that included exposure-based modules. The primary outcome was clinician-assessed PTSD symptom severity (CAPS-5). Secondary outcomes included self-reported symptoms of PTSD, depression, anxiety, and sleep quality. Blood samples were taken to measure levels of drug and eCBs. Overall, PTSD symptoms improved over time. While FAAHi increased AEA levels, there was no effect of FAAHi on PTSD symptoms or any secondary measure. FAAHi combined with internet-delivered CBT did not improve PTSD symptoms to a greater extent than internet-delivered CBT alone. Thus, FAAH inhibition does not appear to be a suitable adjunct treatment for enhancing CBT in PTSD. This study was registered as Eudra-CT 2020-001965-36.

## Introduction

PTSD is a severe mental health condition that can occur after trauma exposure and is characterized by intrusive recollections of trauma-associated memories, avoidance of trauma-related cues or contexts, and hyper-arousal. PTSD has a chronic time course, with only a minority of patients achieving full remission [1]. The gold standard treatment for PTSD is prolonged exposure therapy, a cognitive behavioral therapy (CBT) that uses imaginal and in vivo exposure to trauma-associated stimuli to promote extinction learning [2, 3]. However, efficacy of exposure-based treatments in PTSD is limited by spontaneous return of fear memories, in particular, outside the therapeutic extinction context [1, 4]. Moreover, widespread implementation of exposure-based therapies can be limited by practitioner-, patient-, and system-level barriers [5]. Thus, innovative strategies that promote the efficacy and accessibility of evidence-based PTSD treatments are needed.

Pharmacotherapy could augment the beneficial effects of existing behavioral treatments, but there is little evidence to support the ability of currently available pharmacotherapies to do so [6]. First-line pharmacological treatments commonly prescribed for PTSD, such as selective serotonin reuptake inhibitors, target some symptoms but fail to engage the core underlying pathophysiology of the disorder. As a result, these medications are no more effective when used with exposure therapy than without [7]. As such, there is an unmet need for novel medications with an ability to enhance the effects of behavioral treatment by potentiating extinction of trauma memories.

The endocannabinoid (eCB) system has emerged as a candidate to target the complex symptom profile of PTSD [8, 9]. This system has two primary receptors, CB1 and CB2, both activated by eCB ligands 2-arachidonoyl-glycerol (2-AG) and N-arachidonoylethanolamine (anandamide; AEA) [10–13]. Their activation plays a key modulatory role in fear extinction, stress reactivity, and emotion processing [14, 15]. The primary psychoactive constituent of cannabis, delta-9-tetrahydrocannabinol (THC), is an exogenous activator of CB1 receptors and is often used to reduce feelings of stress, tension, and anxiety [16]. However, exogenous activators such as THC indiscriminately activate CB1 receptors throughout the brain, leading to potentially undesirable effects, including cognitive impairment and appetite dysregulation [17]. A more selective approach to targeting the eCB system for therapeutic purposes is to enhance endogenous AEA signaling through inhibition of its degradation enzyme, fatty acid amide hydrolase (FAAH) [18–21].The availability of AEA is regulated by FAAH [22]. Inhibition of FAAH prolongs synaptic availability of AEA only where and when it is synthesized and released. Thus, FAAH inhibitors amplify the endogenous, physiologically relevant AEA signal [20]. In contrast to THC, FAAH inhibitors are also devoid of psychoactive or rewarding properties, posing little if any abuse liability [18].

Recent studies in humans and animal models suggest that elevated AEA via FAAH inhibition may target the core pathophysiology underlying PTSD [9, 19, 23, 24]. We have shown that FAAH inhibition reduces stress and facilitates fear extinction in healthy humans [19]. Critically, the attenuation of stress via FAAH inhibition does not appear to impair the ability of FAAH inhibition to facilitate extinction memory [19]. This raises the possibility that FAAH inhibition in PTSD may improve outcomes of behavioral treatment in two ways: First, the ability of FAAH inhibition to attenuate stress reactivity may lessen the aversiveness of exposure-based therapy, thus promoting retention in treatment [25]. Secondarily, elevated AEA during exposure therapy may act to promote extinction learning, potentially by strengthening its consolidation. Based on this rationale, we carried out the present study to determine whether FAAH inhibition in combination with internet-delivered exposure-based CBT (iCBT) [26–28] would lead to greater symptom improvements than iCBT alone.

## Methods

In this placebo-controlled, double-blind outpatient trial of the FAAH inhibitor (FAAHi) JNJ-42165279, participants with PTSD were randomized 1:1 to placebo (PBO) or FAAHi for 12 weeks. Participants were recruited via flyers, online/radio advertisement, and referral from healthcare providers and other organizations. The study was conducted at the Center for Social and Affective Neuroscience at Linköping University in Linköping, Sweden from October 2020 to November 2023 and approved by the Swedish Ethical Review Authority (Dnr 2020-57043), the Swedish Medical Products Agency, and registered on Eudra-CT (2020-001965-36).

### Study participants

**Fig. 1:**
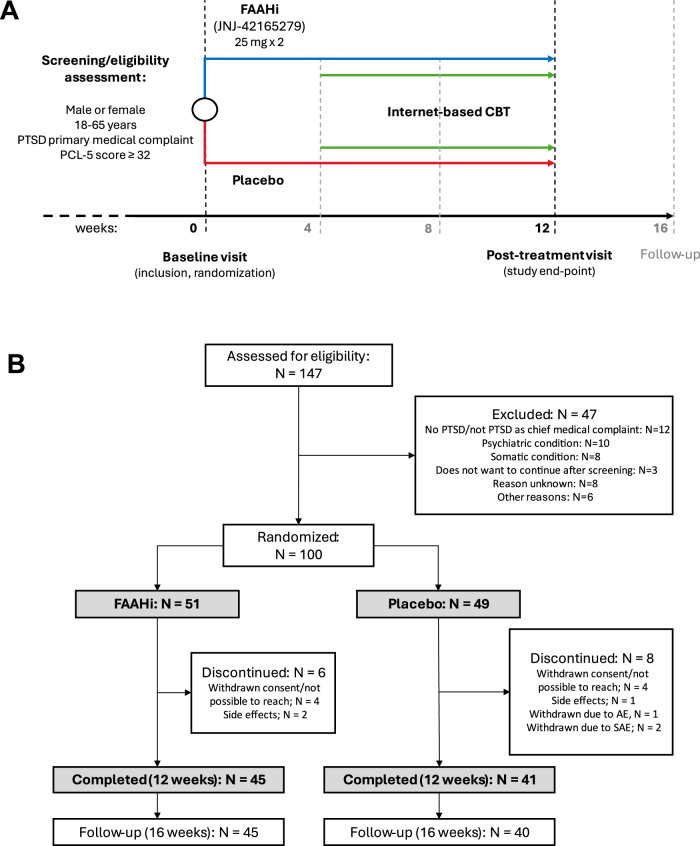
Study schematic and CONSORT diagram. **A** Study schematic depicting study overview. Participants were randomized to FAAH inhibitor (FAAHi) JNJ-42165279 (25 mg, twice daily) or placebo (PBO) for 12 weeks. After week 4, all participants started an 8-week internet delivered cognitive behavioral therapy (CBT) based on exposure learning. Primary outcomes were assessed at week 0 and 12, as well as a 16-week follow-up. Secondary outcomes were assessed at weeks 0, 4, 8, and 12. **B** CONSORT diagram of participant flow.

Potential participants - males and females aged 18–65 years old with PTSD as their primary medical complaint - were first screened via an online platform and then invited for in-person screening. An experienced clinician confirmed presence of a PTSD diagnosis using the Mini International Neuropsychiatric Interview for DSM-5 (MINI). An additional eligibility criterion was a score >32 on the PTSD Checklist for DSM-5 (PCL-5) on screening [29]. Detailed inclusion/exclusion criteria are in Supplemental Materials. At the following visit, informed consent was obtained, and participants were randomized to FAAHi JNJ-42165279, 25 mg b.i.d. or placebo, with medication started the day of inclusion (week 0) and ended at week 12. Randomization was stratified by sex. All participants received iCBT as psychological treatment for PTSD [26, 28], from week 5 to 12 of medication treatment. A follow-up phone visit occurred at 16 weeks. See Fig. 1 for study schematic.

### Drug

The FAAHi JNJ-42165279, provided by Janssen Research & Development for use in this investigator-initiated trial, is a potent, selective, and orally bioavailable inhibitor of the FAAH enzyme [30] previously studied in Phase 1 and 2a studies [21, 31]. JNJ-42165279 tablets (25 mg) and matched placebo were provided to the Linköping University Hospital Pharmacy by a contractor with blinded labels. All study staff were blinded through the entirety of the study, and only unblinded following completion of the last participant and locking of the final database. Randomization and blinding were insulated from investigators. Randomization was carried out by an independent statistician at the clinical trials support service of Linköping University Hospital (Forum Östergötland). The allocation sequence was generated using Procedure Blockrand in R, with a block size of 6, in a manner concealed from all study staff. The sequence remained concealed until the study was completed, data were locked, and codes were broken. Medication vials labeled with the blinded randomization numbers were delivered to research nurses, who had no access to any additional information. Research nurses enrolled participants, and dispensed medication according to the respective randomization number. Medication was self-administered and adherence was assessed by plasma drug concentrations and pill-count. The hospital pharmacy was contracted for potential emergency code-breaking procedures; three code breaking events occurred, one due to mild but multiple adverse events (AE) and two due to serious adverse events (SAE).

### Internet-based cognitive behavioral therapy (iCBT)

The iCBT was based on a manual tailored for treating PTSD, including validated components such as psychoeducation, anxiety coping skill training, exposure (including both in vivo and imaginal exposure), and cognitive restructuring [26, 32, 33]. Research suggests that guided iCBT can be as effective as face-to-face CBT in the treatment of mild to moderate disorders [34]. Here, iCBT consisted of eight text-based modules delivered once a week over 8 weeks (weeks 5–12 of medication treatment) and included practice homework. Supervision and support during the trial was provided online on a weekly basis by one of two experienced clinical psychologists. Adherence was evaluated by number of sessions completed, and total time of therapist engagement throughout iCBT was documented. This trial was conducted during the COVID-19 pandemic, and the use of internet-delivered therapy allowed delivery of an evidence-based therapeutic intervention, while minimizing exposure risk to patients and providers.

### Data collection

Demographic information was collected via self-report. Week 0 consisted of an inclusion and baseline visit. Participants provided safety and efficacy measures at weeks 4, 8, and 12. All primary and secondary outcomes were assessed at week 0 and 12; secondary outcomes PCL-5 and Pittsburgh Sleep Quality Index (PSQI) [35] were also assessed at weeks 4 and 8. A phone visit occurred at week 16 for safety follow-up and additional measures. Adverse event (AE) information was systematically collected in weeks 0–12.

### Measures

The primary outcome was PTSD symptom severity as assessed via CAPS-5, a structured interview corresponding to the DSM-5 PTSD criteria [36], performed by trained research nurses. CAPS-5 scores range from 0 to 80. The secondary outcomes included self-reported symptoms of PTSD assessed with PCL-5 [37] (score range 0–80), symptoms of anxiety and depression assessed with the Comprehensive Psychopathological Rating Scale, Self-Administered (CPRS-S-A) [38] (anxiety subscale: 0–24; depression subscale: 0–27) and sleep quality rated with PSQI [3] (score range 0–21). For all self-report questionnaires, higher values indicate greater impairment.

### Biochemical analyses

#### Endocannabinoid analysis

Plasma levels of AEA and 2-AG were determined via liquid chromatography with tandem mass spectrometry (LC/MS/MS), as previously described [19, 39].

#### Drug levels

Plasma concentrations of JNJ-42165279 were measured using LC/MS/MS as done previously [21] and described in detail in Supplemental Materials.

### Statistics

#### Sample size calculation

A published meta-analysis reported a pooled effect size of Cohen’s d = 0.81 for specific psychological PTSD treatments compared to non-specific supportive treatment [40]. Based on a more conservative effect size, d = 0.60, 100 participants randomized 1:1 to active treatment or placebo yields a power of 85% to detect this effect size or greater with a two-tailed α = 0.05.

#### Analysis

Efficacy analyses are based on an intent-to-treat (ITT) analysis, consisting of all randomized participants receiving at least one medication dose (*N* = 100).

Continuous variables were evaluated for normality and homogeneity of variance, and group comparisons were carried out using Student’s *t* test, or, if assumptions were violated, using the non-parametric Mann–Whitney U test. Categorical variables were evaluated with Chi-square test and Fisher’s exact test used when applicable. In all analyses, a two-tailed probability level of 0.05 or lower was considered significant. Spearman’s rank order correlations between CAPS-5 and PCL-5 scores over the 12-week study period were used to determine if the clinician-assessed scores reflected the self-report scores, due to the skewed distribution of the data. Similarly, Spearman’s rank order correlations were used to determine the association between JNJ-42165279 and AEA-levels to explore target engagement.

Constrained linear mixed models (CLMMs) were applied to evaluate drug effect on primary and secondary outcomes. Treatment group and time were included as fixed effects and individual identity as random effect. Gender-specific time and treatment effects were additionally examined. Residual graphic diagnostics were applied for detecting violation of model assumptions. As a sensitivity analysis, treatment effect was examined by adjusting baseline score in a linear regression model for each outcome.

## Results

### Study population

Participants (*N* = 100) were assigned to JNJ-42165279 (FAAHi, *n* = 51) or placebo (PBO, *n* = 49; Fig. 1). Participants were mostly female (85%) with mean age 38 ± 12 years. No differences between treatment groups regarding demographic or baseline clinical characteristics were found (Table 1). Scores for primary and secondary outcome measures at all timepoints are in Supplementary Table S3.

**Table 1:** Demographic and baseline clinical characteristics.

	Overall *N* = 100	PBO *N* = 49	FAAHi *N* = 51	*p*-value^a^
Sex: female	85 (85%)	43 (88%)	42 (82%)	0.45
Age	38 (12)	37 (12)	39 (12)	0.52
Body mass index	24.6	24.4	25.0	0.7
Occupation				
Work	51 (51%)	26 (53%)	25 (49%)	0.69
Studies	22 (22%)	9 (18%)	13 (26%)	0.39
Unemployed/sick leave/retired	27 (27%)	14 (29%)	13 (26%)	0.73
Current psychoactive medication^b^	58 (58%)	29 (59%)	29 (59%)	1.00
Current psychiatric co-morbidity^c^	53 (53%)	28 (57%)	25 (49%)	0.42
PTSD diagnosis (CAPS-5)	81 (81)	40 (82)	41 (80)	0.87
PTSD severity score (CAPS-5)	31.8 (8.1)	32.5 (9.0)	31.1 (7.1)	0.41
PTSD exposure type (CAPS-5)				
Life threatening	56 (56%)	24 (49%)	32 (63%)	0.12
Serious injury	38 (38%)	17 (35%)	21 (41%)	0.48
Sexual assault	52 (52%)	29 (59%)	23 (45%)	0.36
Self-reported PTSD symptoms (PCL-5)	44.3 (12.2)	45.2 (13.6)	43.4 (10.8)	0.45
Anxiety symptoms (CPRS-S-A)	10.8 (4.1)	10.8 (4.4)	10.8 (3.9)	0.97
Depressive symptoms (CPRS-S-A)	10.4 (4.1)	10.6 (4.5)	10.3 (3.7)	0.76
Sleep quality (PSQI)	11.0 (3.7)	11.4 (3.9)	10.6 (3.5)	0.32
Childhood trauma (CTQ)	54.0 (20.6)	57.6 (23.5)	50.8 (16.9)	0.11

Data presented as mean (SD) for continuous measures, and *n* (%) for categorical measures.

*CAPS-5* Clinician-Administered PTSD Scale for DSM-5, *PCL-5* PTSD Check List for DSM-5, *CPRS-S-A* Comprehensive Psychopathological Rating Scale, Self-Administered, *PSQI* Pittsburgh Sleep Quality Index.

^a^Student’s *t* test for continuous variables, Chi-square for categorical variables.

^b^Including regular use and/or as needed.

^c^Any current co-morbid psychiatric disorder according to MINI.

At 12 weeks, 14 participants had dropped out (*n* = 14 female). Dropouts were younger (32 ± 11 years) compared to completers (39 ± 12 years) (t[98] = 2.19, *p* = 0.031). There were no other differences between the *n* = 6 FAAHi and *n* = 8 PBO dropouts across any measure.

### Primary outcome: clinician-assessed PTSD severity

**Fig. 2:**
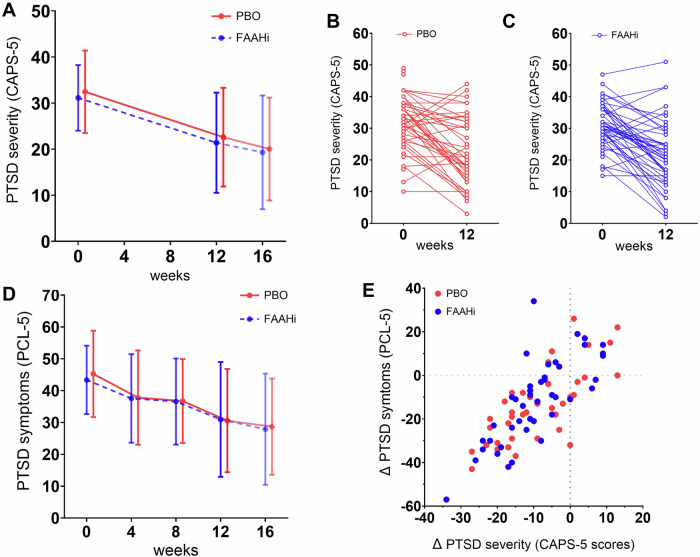
Clinician-assessed and self-reported PTSD symptoms decreased over time but were unaffected by FAAH inhibition. **A** Mean scores at week 0 (baseline), week 12 (post-treatment) and week 16 (phone follow-up) for FAAHi and PBO treatment groups. No drug effect was observed, only an effect of time. Error bars represent standard deviation. **B** Individual CAPS-5 scores at week 0 and 12 for the PBO group. **C** Individual CAPS-5 scores at week 0 and 12 for the FAAHi group. **D** Mean self-reported PTSD symptoms as measured by PCL-5 at week 0, 4, 8 and 12 and 16 for PBO and FAAHi treatment arms. No drug effect was observed. Error bars represent standard deviation. **E** The change in CAPS-5 scores between week 0 and 12 correlated strongly to the change in PCL-5 scores between week 0 and week 12, in both treatment groups.

In the CLMM, there was a significant main effect of time, such that PTSD severity, assessed by CAPS-5, decreased between weeks 0 and 12 in both groups (PBO: −8.6 ± 10.5, FAAHi: −9.6 ± 10.1; β = −8.8; 95% CI: −11.94, −5.72; *p* < 0.001; Fig. 2A–C). There was no significant main effect of drug (β = −1.06; 95% CI: −5.27, 3.15; *p* = 0.617).

### Secondary outcome: self-reported PTSD symptoms

Self-reported PTSD symptoms measured by PCL-5 decreased from week 0 to 12 in both groups (PBO: −13.7 ± 16.3, FAAHi: −12.7 ± 17.8; β = −13.7; 95% CI: −18.8, −8.6; *p* < 0.001; Fig. 2D), with no effect of drug (β = 0.73; 95% CI: −6.15, 7.60; *p* = 0.834). At week 16, further attenuation in self-reported symptoms was observed (PBO: −4.2 ± 12.5, FAAHi: −2.8 ± 8.8, Fig. 2D).

Change in self-reported (PCL-5) and clinician-assessed (CAPS-5) PTSD symptom scores from week 0 to 12 were highly correlated (PBO: r_s_[39] = 0.73, *p* < 0.001, FAAHi: r_S_[43] = 0.82, *p* < 0.001; Fig. 2E).

### Other secondary outcomes: self-reported anxiety, depression, sleep quality

**Fig. 3:**
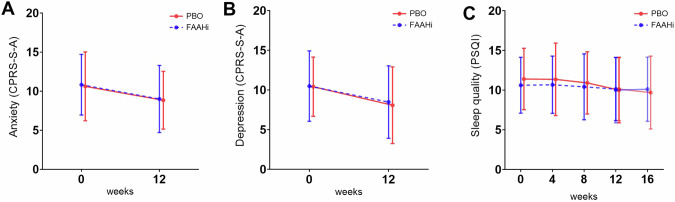
FAAH inhibition did not improve any secondary measure. Mean scores of secondary outcome measures for the PBO and JNJ treatment groups. No effect of the drug was observed for any of these outcome measures. Error bars represent standard deviation. **A** Anxiety levels (CPRS-S-A) at week 0 and week 12. **B** Depressive symptoms (CPRS-S-A) at week 0 and week 12. **C** Sleep quality (PSQI) at week 0, 4, 8, 12 and 16.

Secondary outcomes decreased over the 12-week study period in both groups, including anxiety (PBO: −1.8 ± 4.1, FAAHi: −1.3 ± 3.9; β = −1.56; 95% CI: −2.75, −0.37; *p* = 0.011; Fig. 3A) and depressive symptoms (PBO: −2.1 ± 4.6, FAAHi: −2.1 ± 4.6; β = −2.04; 95% CI: −3.5, −0.62; *p* = 0.005; Fig. 3B). There was no effect of drug on anxiety (β = −0.25; 95% CI: −1.77, 1.28; *p* = 0.75) or depression: (β = −0.21; 95% CI: −2.08, 1.65; *p* = 0.82). Sleep quality evaluated by PSQI scores remained stable between week 0 and 12 (PBO: −1.4 ± 3.5, FAAHi: −0.4 ± 3.3; β = 0.77; 95% CI: −0.79, 2.32; *p* = 0.33; Fig. 3C). For complete details on the CLMM, see Supplementary Table S4.

### Sensitivity and additional analyses

There was no main effect of sex on any primary or secondary outcome (*p* > 0.2) and adjusting for baseline scores did not influence results (Supplementary Table S5). There was also no effect of baseline AEA on the primary outcome (Supplementary Table S6). There were no effects of FAAHi on any specific PTSD symptom cluster (Supplementary Table S2; Supplementary Fig. 2).

### iCBT

Engagement in iCBT was equivalent in the two groups (median [IQR] completed sessions; PBO: 7.0[3.0, 8.0], FAAHi: 6.0[3.0, 7.5]; *U* = 1153, *p* = 0.50). No correlations were found between the number of completed iCBT sessions, therapist time spent, and any primary or secondary outcome. There was also no difference between treatment groups in subjective units of distress following therapy modules (see Supplementary Results and Supplementary Fig. S1 for details).

### Adherence

Adherence using pill count at the 12-week post-treatment visit as an approximation was high overall, with no difference between treatment arms (median [IQR]; PBO: 10.0[6, 16], FAAHi: 8 [4, 13], *U* = 779.5; *p* = 0.22, dropouts excluded).

**Fig. 4:**
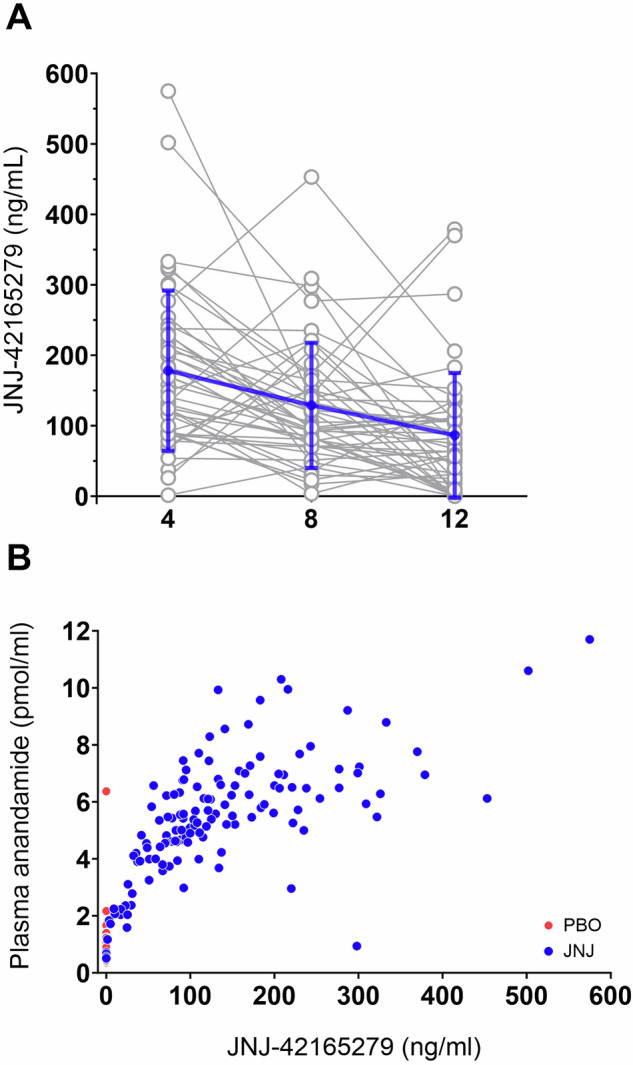
FAAH inhibitor JNJ-42165279 was detectable in blood and correlated with anandamide levels. **A** Individual plasma concentrations of JNJ-42165279 at week 4, 8 and 12 in the JNJ treatment arm. Many participants took the study drug the same day as the blood sample was drawn, which is why results should be used as an indicator of compliance and not trough levels. **B** Plasma concentrations of JNJ-42165279 correlated strongly with anandamide levels.

No participant in the PBO group had a detectable level of JNJ-42165279. Plasma concentrations of JNJ-42165279 in the FAAHi group declined from 178 ± 114 (*n* = 47) at week 4, to 129 ± 89 (*n* = 44) at week 8 and then further dropped to 86 ± 88 (*n* = 45) at week 12 (Fig. 4A). We found no correlations between CAPS-5 scores and plasma concentrations of JNJ-42165279 at week 8 (r_s_[23] = 0.25, *p* = 0.24) nor at week 12 (r_s_[28] = 0.042, *p* = 0.83).

### Effect of JNJ-42165279 on anandamide levels

Plasma concentrations of JNJ-42165279 correlated strongly with AEA levels at the corresponding time point (r_s_[133] = 0.67, *p* < 0.001, FAAHi group only; see Fig. 4B) confirming expected target engagement.

### Safety

Most participants reported at least one AE, however, most were mild (PBO: *n* = 148/181 [82%], FAAHi: *n* = 135/169 [80%]) to moderate (PBO: *n* = 31/181 [17%], FAAHi: *n* = 31/169 [18%]) with no difference in severity between groups (mild: *p* = 0.77, moderate: *p* = 0.78, severe: *p* = 0.68). One participant was withdrawn due to mild but multiple AEs and two due to occurrence of SAEs; all in the placebo arm. AEs occurring in >5% of participants in any treatment arm are reported in Supplementary Table S7 (for all AEs see Supplementary Tables S8).

### Bayesian analysis of primary outcome

A minimal clinically meaningful difference (MCMD) for the CAPS has previously been reported to be in the range of 0.5–0.8 SD [41]. In our study, with a pooled SD of 10.8, this corresponds to a 5.4–8.6 CAPS unit difference. Using the lower bound of this interval, a Bayesian analysis provides very strong evidence against a clinically meaningful treatment effect [likelihood under H_0_: 0.00116; likelihood under H_1_: 0.998840; Bayes Factor (BF_10_): 864.42]. Even for a difference lower than the MCMD, e.g. 3 units, the analysis provides strong evidence against its presence, with a BF = 18.52. This analysis makes it highly unlikely that a meaningful treatment effect exists, but was missed, e.g. due to insufficient power, or random variation.

## Discussion

Evidence in preclinical models and healthy humans has highlighted a role for CB1 receptors and AEA in fear extinction [9, 19, 23, 42, 43]. As extinction learning forms the foundation for exposure-based CBT, our goal was to determine if elevating AEA together with exposure-based CBT would be more effective than CBT alone. While PTSD symptoms did significantly improve over time in both treatment groups, there was no effect of FAAH inhibition on PTSD symptoms, despite the fact that the drug was confirmed to elevate AEA levels. We also found no effect of FAAHi on any secondary measure, including self-reported symptoms of PTSD, anxiety, depression, or sleep quality.

Previous studies exploring FAAHi as a therapeutic intervention have shown modest results. The inhibitor used here showed some evidence of an anxiolytic effect in participants with social anxiety disorder, but did not meet the primary endpoint [21]. Subsequent analyses found that the selected dose (25 mg q.d.) may not have been high enough to maintain the level of FAAHi needed to reduce clearance of AEA at trough. Indeed, drug PK and AEA level analysis suggested that participants with drug and AEA levels in the upper tertile did experience a greater symptom reduction. Based on these findings and PK/PD modeling, we doubled our dose to 25 mg b.i.d. Despite this, we found no effect of FAAHi on PTSD symptoms. Analyses covarying for AEA or drug levels failed to show a significant effect. This same dose also recently failed to meet its primary outcome in a study of adolescents and adults with autism spectrum disorder, though small-to-moderate effects were reported on some secondary outcomes [44].

A possible reason for the failure to detect a drug effect is that the efficacy of our iCBT intervention may have resulted in a ceiling effect. However, we consider this to be unlikely for multiple reasons. First, for the first four weeks of the study, drug was administered in the absence of iCBT specifically to allow an evaluation of potential drug effects in the absence of behavioral treatment. Despite this, between-group differences were not evident on outcome measures, including the secondary outcome of self-reported PTSD symptoms, which was highly correlated with clinician-assessed PTSD measurements at weeks 0 and 12. Thus, one month of ‘FAAHi only’ had no advantage over ‘placebo only’. Furthermore, a recently completed, yet unpublished multi-center, randomized, controlled clinical trial compared another FAAHi, JZP150 to placebo in the absence of any psychotherapy. It was recently reported via press release that JZP150 was ineffective in improving PTSD symptoms over 12 weeks [45]. Collectively, available data suggest that FAAHi, whether administrated alone or with psychotherapy, does not reduce PTSD symptoms.

A potential limitation is our decision to use iCBT instead of Prolonged Exposure (PE), the gold standard PTSD treatment. However, we selected iCBT as our approach for several reasons. First, while PE is the gold standard for PTSD, it is highly resource demanding, resulting in a clinical bottleneck due to lack of available trained therapists [5]. This makes it difficult to deploy on a large scale and limits accessibility. It also requires patients to regularly commute to the clinic, which was particularly challenging during the COVID-19 pandemic, when this study was completed. Moreover, meta-analytic evidence suggests that iCBT may be as effective as face-to-face CBT in the treatment of mild to moderate disorders [34]. Finally, given the easier clinical implementation, but potentially lower efficacy of iCBT vs PE, a medication that could potentiate iCBT effects would be a particularly attractive combination to reach a large patient population with minimal burden to patients or clinical resources.

Our study was initiated based on converging data from animal models and human experiments consistently supporting the notion that elevating AEA via FAAHi might be an efficacious intervention for PTSD (for review, see ref. [9]). This conclusion was scaffolded by extensive literature that identified a critical role for CB1 receptors in fear extinction and stress response buffering [14] and human behavioral and imaging results with THC, a partial CB1 agonist [46, 47]. It was further supported by our own previous work in healthy humans, showing that FAAHi enhanced fear extinction when tested 24 h after fear acquisition and attenuates subjective and autonomic stress reactivity, as well as stress-induced negative emotions [19]. Exploring the relationship between laboratory models of fear conditioning and PTSD symptoms could provide insight into the predictive validity of these measures. However, a recent review suggests that drug effects to facilitate fear extinction in animals can translate to healthy humans, and sometimes even patients with PTSD; however, even then, these compounds are not successful in improving clinical PTSD symptoms [48]. This suggests a need for considering how outcomes from lab-based models of fear conditioning relate to real-world behaviors in patients with PTSD.

Our previous study in healthy volunteers used PF-04457845, an irreversible FAAH inhbitor [19]. In contrast, JNJ-42165279 is slowly reversible [30]. It was previously shown to dampen limbic responses during an emotional faces task, but had no effect on within-session extinction learning in healthy adult males [49]. It could be speculated that the reversible nature of FAAH inhibition by JNJ-42165279 accounts for this lack of effect. However, our previous study with PF-04457845 also failed to show a significant effect on within-session extinction, and was only effective in promoting fear extinction when tested 24 h after extinction [19]. As our iCBT treatment took place over several weeks with drug on board for the duration, we still expected to see the therapeutic effects of enhanced extinction learning, as evidenced by greater efficacy of the iCBT intervention. Thus, it could be speculated that, in clinical populations, the partial agonist activity of AEA is insufficient to robustly influence symptoms of pathological fear. If so, a more effective approach could be to increase levels of 2-AG, which acts as a full CB1 agonist, via inhibition of its degrading enzyme, monoacylglycerol lipase (MAGL) [9, 50]. Recent translational evidence suggests that deficient 2-AG signaling can promote fear generalization [51]. However, chronic administration of MAGL inhibitors can lead to desensitization of CB1 receptors [52], suggesting that dosing and duration of administration would need to be carefully considered.

Two important alternative interpretations of our data are possible. First, impaired ability to extinguish fear memories is thought to be a core symptom of PTSD, leading to the expectation that drugs with an ability to promote fear extinction under laboratory conditions would be clinically beneficial [8]. It is, however, possible that the pathological fear memories in PTSD are generalized, over-consolidated, or both, in a manner not captured by laboratory assessments of fear learning and extinction in healthy people. Secondly, while FAAHi treatment was able to elevate AEA levels in our clinical population to an extent similar to that previously observed in healthy controls [19], our findings suggest that disrupted CB1 signaling may be part of PTSD pathophysiology, preventing the expected beneficial effects of elevated ligand levels.

A limitation of this study is that 85% of participants were female, and the study was not adequately powered to detect sex or gender-specific effects. Preclinical literature has found that manipulations of both the CB1 receptor and FAAH can influence anxiety and fear extinction in males, but more recent evidence suggest that these manipulations produce incongruent or null effects in females [53–56]. Thus, the possibility exists that our results could have been influenced by a sample enriched in females. Our previous studies in healthy humans assessing genetic variation in AEA levels or pharmacological elevation did not produce gender-specific effects, though again, these studies were not powered to directly assess this potential interaction. However, recent cross-sectional data suggests that AEA levels maybe decreased in men, but not women, with PTSD [57]. Future studies systematically exploring sex- and gender-based differences in eCB function and related behaviors will be critical in determining the translational validity of these findings and subsequently developing novel pharmacotherapies more broadly.

Additional features of our sample and study design could have impacted our outcomes. For instance, we have only cursory information on trauma exposure, and more specific information, such as age at trauma or time since trauma, could be relevant to include for future studies. Further, while we predicted that FAAHi could reduce the aversiveness of the therapy, it is possible that this could also attenuate the efficacy of the therapy. However, we saw no effect of drug on ratings of distress during completing the therapy. Overall, we found that FAAHi did not enhance the response to iCBT for PTSD in our sample of primarily women. Future research should consider investigating whether eCB function is indeed altered in PTSD, and how this may be related to fear memory processes.

## Supplementary information

Supplementary Materials

## Data Availability

Data are not publicly available due restrictions from the local ethical committee but are available from the corresponding author on reasonable request.
