# Supplementary material for: The efficacy of elevating anandamide via inhibition of fatty acid amide hydrolase (FAAH) combined with internet-delivered cognitive behavioral therapy in the treatment of post-traumatic stress disorder: a randomized, placebo-controlled clinical trial
Source: Neuropsychopharmacology. 2025 May 17;50(10):1564–72. doi: 10.1038/s41386-025-02128-w (PMC12339700; doi:10.1038/s41386-025-02128-w)
Supplement: Supplementary file 1 — Supplementary Materials [file 41386_2025_2128_MOESM1_ESM.docx]

**SUPPLEMENTARY MATERIALS**

Contents

[SUPPLEMENTAL METHODS 2](#_Toc196916348)

[Detailed Inclusion and Exclusion Criteria 2](#_Toc196916349)

[Inclusion Criteria: 2](#_Toc196916350)

[Exclusion Criteria 3](#_Toc196916351)

[Drug 6](#_Toc196916352)

[Internet-based cognitive behavioral therapy (iCBT) 6](#_Toc196916353)

[Data collection 7](#_Toc196916354)

[Analysis of JNJ-42165279 7](#_Toc196916355)

[Secondary outcomes: PSQI Scoring 8](#_Toc196916356)

[**Table S1.** Revised scoring of PSQI item 4 – hours of actual sleep. 8](#_Toc196916357)

[SUPPLEMENTAL RESULTS 9](#_Toc196916358)

[**Results:** Analysis of JNJ-42165279 9](#_Toc196916359)

[**Results**: Subjective units of distress 9](#_Toc196916360)

[**Results:** PTSD symptom clusters 10](#_Toc196916361)

[**Table S2.** CAPS-5 subscales of PTSD symptom clusters 11](#_Toc196916362)

[**Table S3.** Primary and secondary outcomes at each timepoint 12](#_Toc196916363)

[**Table S4:** Effect of JNJ-42165279 on primary and secondary outcomes 13](#_Toc196916364)

[**Table S5:** Treatment effect after adjusting for Baseline (week 0) scores 14](#_Toc196916365)

[**Table S6:** Treatment effect after adjusting for baseline (week 0) AEA 14](#_Toc196916366)

[**Table S7:** Adverse events 14](#_Toc196916367)

[**Table S8:** All treatment emergent adverse events by System organ class and preferred term. 16](#_Toc196916368)

# SUPPLEMENTAL METHODS

## Detailed Inclusion and Exclusion Criteria

### Inclusion Criteria:

1. Age >18 and <65 years.
2. Diagnosed with current PTSD according to the *Diagnostic and Statistical Manual of Mental Disorders*-5 DSM-5, as determined by The Mini International Neuropsychiatric Interview, MINI-7.0 for DSM-5 and clinical examination.
3. Has a PCL-5 score ≥32
4. Reports PTSD-related symptoms as primary medical complaint
5. Willing to provide informed consent and comply with study procedures.
6. Able to self-administer study medication and swallow it whole with water
7. Able to speak and read Swedish.
8. Has access to computer, tablet or other device enabling interaction with internet-based intervention.
9. To be eligible, women must have a negative serum or urine pregnancy test prior to the start of study drug, and either not be of childbearing potential, or agree to use a method of contraception that is highly effective for the duration of the study and for 4 weeks after receiving the last dose of study medication. Not of childbearing potential is defined as postmenopausal (>45 years of age with amenorrhea for at least 12 months, or any age with amenorrhea for at least 6 months and a serum follicle stimulating hormone (FSH) level >40 IU/L); permanently sterilized (e.g., tubal occlusion, hysterectomy, bilateral salpingectomy); not heterosexually active in the past 12 months and not having an intent of becoming heterosexually active in the coming 12 months; or otherwise incapable of pregnancy.
10. Subjects must be otherwise healthy for their age group or medically stable with or without medication, as determined by physical examination, medical history, vital signs, and 12-lead ECG performed at the eligibility assessment visit or at the baseline visit. If there are abnormalities, they must be consistent with any known, underlying condition of the participant that is stable under treatment; or they must be without clinical significance. This determination will be made by the Investigator or designee, aided by specialist consults as needed, and documented.
11. Subjects must be otherwise healthy or medically stable on the basis of clinical laboratory tests performed at the eligibility assessment visit. If the results are outside the normal reference ranges, the subject may be included only if the abnormalities or deviations from normal are deemed not to be clinically significant. This determination will be made by the Investigator or designee, aided by specialist consults as needed, and documented.

### Exclusion Criteria

1. A lifetime history of any major psychiatric disorder that, in the judgment of the Investigator, based on MINI 7.0 interview, clinical examination and any other available clinical information, limits or may come to limit the ability of the subject to provide informed consent, comply with study procedures, or safely participate in the study; including, but not limited to schizophrenia or other chronic psychotic disorder, or bipolar disorder.
2. A current psychiatric condition that, in the judgment of the investigator, may limit the ability of the subject to comply with study procedures or put subject at unacceptable risk; including but not limited to psychotic depression, severe eating disorder, borderline personality disorder or severe obsessive-compulsive disorder.
3. A current cognitive impairment that could interfere with the ability of a subject to provide informed consent or comply with study procedures, as indicated by a score of 24 or lower on the Mini Mental State Examination – Swedish Revision (Swedish: Mini Mental State Examination. Svensk Revidering, MMSE-SR).
4. A current clinical diagnosis of severe (but not mild or moderate) DSM-5 alcohol use disorder, as determined by the Investigator or designee based on MINI 7.0 interview, clinical examination, and evaluation of the biomarker serum phosphatidyl ethanol (PEth); or current clinically significant alcohol withdrawal syndrome.
5. A current clinical diagnosis of moderate or severe DSM-5 substance use disorder, other than nicotine, as determined by the Investigator based on MINI 7.0 interview, clinical examination
6. Any presence of an illicit substance on eligibility assessment visit urine toxicology screen
7. A current or recent (within past 6 months) history of clinically significant suicidal ideation, corresponding to a score of 4 (active suicidal ideation with some intent to act, without specific plan) or 5 (active suicidal ideation with specific plan and intent) for ideation on the Columbia Suicide Severity Rating Scale (CSSR-S ), or a history of suicidal behavior within the past year. Subjects with a prior suicide attempt, or prior serious suicidal ideation/plan > 6 months ago will be carefully screened for current suicidal ideation and only included at the discretion of the Investigator.
8. Insufficient memory of the trauma (for prolonged exposure to be effective).
9. Ongoing traumatization
10. A dissociative disorder which is more severe or affects the subject more than their PTSD.
11. Any ongoing specific psychological treatment for PTSD.
12. Current or past thyroid disease or dysfunction that is not currently stable in treatment or monitored. Subjects treated for thyroid disease may be enrolled following review of their records of diagnosis and treatment history at the discretion of the Investigator or designee, aided by specialist consult as needed, to ensure disease/treatment stability and compliance.
13. Any abnormal finding on a 12-lead ECG, unless not clinically significant in the judgment of the Investigator or designee, aided by adequate specialist consults.
14. A history of or current liver or renal insufficiency; clinically significant cardiac, vascular, pulmonary, gastrointestinal, endocrine, hematologic, rheumatologic, psychiatric, or metabolic disturbances (e.g. unstable situation needing monitoring or regular dose adaptations).
15. A history of malignancy within 5 years before eligibility assessment (with the exception of squamous and basal cell carcinomas of the skin and carcinoma in situ of the cervix, or malignancy that in the judgment of the Investigator, aided by specialist consults as needed, is considered cured with minimal risk of recurrence).
16. A history of positive tests for hepatitis B surface antigen (HBsAg) or hepatitis C antibody (anti-HCV) positive, or other clinically active liver disease, or tests positive for HBsAg or anti-HCV at eligibility assessment; unless successfully treated as shown by RNA / DNA tests, or due to previous vaccine exposure.
17. A history of human immunodeficiency virus (HIV) antibody positive, or tests positive for HIV at eligibility assessment.
18. Major surgery, (e.g. requiring general anesthesia) within 8 weeks before eligibility assessment, or will not have fully recovered from surgery, or has surgery planned during the time the subject is expected to participate in the study or within 4 weeks after the last dose of study drug administration. Subjects with planned surgical procedures to be conducted under local anesthesia may participate.
19. History of spontaneous, prolonged or severe bleeding, or has donated one or more units (approximately 450 mL) of blood or had acute loss of an equivalent amount of blood within 90 days prior to study drug administration.
20. Any other condition due to which, in the judgment of the Investigator, participation would not be in the best interest of the subject or that could prevent, limit, or confound the protocol-specified assessments.
21. Pregnancy, breast-feeding, or planning to become pregnant.
22. People with counter-indications to MRI scanning (metal in body, claustrophobia) or task completion (i.e. severe, uncorrected vision or hearing impairments) who are otherwise eligible and provide consent will be excluded from the MRI session, but will otherwise be able to participate.
23. Subject has received an investigational drug or used an investigational medical device within 3 months before the planned start of study or is currently enrolled in an investigational study.
24. Subject is an employee of the investigator or study site, with direct involvement in the proposed study or other studies under the direction of that investigator or study site; or is a family member of the employees or the investigator.

## Drug

The FAAH inhibitor JNJ-42165279, provided by Janssen Research & Development for use in this trial, is a potent, selective, and orally bioavailable mechanism-based inhibitor of the FAAH enzyme (28). This compound was previously studied in multiple Phase 1 studies, including a positron emission tomography study (29). It was also used in Phase 2a studies in patients with social anxiety disorder (20) and major depressive disorder with anxious distress (currently unpublished; results posted on clinicaltrials.gov NCT02498392) treated with 25mg q.d., as well as a phase 2a study in autism spectrum disorder (submitted: NCT03664232), which increased from 25mg once daily (q.d.) to 25mg twice daily (b.i.d.).

JNJ-42165279 tablets (25 mg) and visually indistinguishable placebo tablets were provided to the Hospital Pharmacy at Linköping University Hospital by a contractor, Clinical Supplies Management Europe SA, as patient-ready packs with blinded labels. Randomization and blinding were insulated from any of the investigators. Medication was self-administered and in total five bottles of 35 pills each (total 175 pills; full drug adherence throughout the study required 168 pills) were dispersed at in-person sessions conducted at 4-week intervals. Adherence was assessed by assessment of plasma drug concentrations and pill-count at week 4, 8, and 12 of dosing. The hospital pharmacy was contracted for potential emergency code-breaking procedures; three code breaking events occurred, one due to mild but multiple adverse events (AE) and two due to serious adverse events (SAE).

## Internet-based cognitive behavioral therapy (iCBT)

The iCBT was based on a manual tailored for treating PTSD, including validated components commonly used in CBT for PTSD, such as psychoeducation, anxiety coping skill training, exposure, and cognitive restructuring (25,30,31). Previous research suggests that guided iCBT can be as effective as face-to-face CBT in the treatment of mild to moderate disorders (32). It also provides a more accessible intervention than required by in-person therapy. The iCBT in this study consisted of eight text-based modules delivered once a week over a period of 8 weeks (weeks 5 to 12 of medication treatment) and also included homework to practice during the weeks. Supervision and support during the trial was provided online on a weekly basis by one of two experienced clinical psychologists specialized in PTSD and involved in the study. Adherence with iCBT was evaluated by the number of sessions completed, based on the therapist’s judgement of whether the participant seemed to have acquired the central knowledge from each respective session. In addition, the total time of therapist engagement with each participant throughout the eight weeks of iCBT was documented.

## Data collection

Demographic information was collected by self-report on the online screening platform. Eligible participants completed an inclusion and baseline visit (week 0). Participants provided safety and efficacy measures at 4, 8, and 12 weeks. All primary and secondary outcomes were assessed at week 0 and 12; and two of the secondary outcomes (PCL-5, PSQI) were additionally assessed at week 4 and 8. An additional phone and online visit was scheduled for week 16, for safety follow-up and additional measures.

Adverse event (AE) information was systematically collected at visits 3 through 6 via laboratory tests, assessments of blood pressure and heart rate, and questions about general well-being and psychiatric symptoms. Participants were also encouraged to contact a study nurse if they suspected the occurrence of an AE between visits.

## Analysis of JNJ-42165279

The plasma concentrations of JNJ-42165279 were measured using validated liquid chromatography methods with tandem mass spectrometry (LC‑MS/MS) detection (Turbo-ionspray in positive mode), 6500 series mass spectrometer (Sciex, Framingham, USA) after protein precipitation with acetonitrile over an Ostro filter plate (Waters, Milford, USA). For chromatographic separation an Agilent 1290 series LC system (Agilent, Diegem, Belgium) was used. The column (Waters UPLC BEH C18 30*2.1 mm, 1.7µm, Waters) was kept at a temperature of 60 °C. The mobile phase consisted of 10 mM ammonium formate (pH 4) in water (A) and acetonitrile (B). The gradient ran from ratio (A/B) 73/27 to 69/31 (% v/v) in 0.6 minutes, followed by a column flush step at 5/95 (% v/v) for 0.23 min. The flow rate was 1.5 mL/min. A stable isotope labeled analogue (^2^H_8_) of JNJ-42165279 was used as internal standards. For the MS/MS detection, the mass transitions (m/z) of JNJ-42165279 and internal standard were 411.1 → 257.2 and 419.1 → 265.2 respectively. The internal standards were used to calculate peak area response ratios that were used for quantification (linear regression using weighting factor 1/x^2^). The validated concentration range was 1.00 – 1000 ng/mL. The descriptive statistics of the QC samples showed that the interbatch precision was ≤4.8%, whereas the interbatch bias was within the range of 0.0% to 3.0%. Stability of JNJ-42165279 in plasma samples was demonstrated for up to 27 hours at room temperature, 896 days at -70°C, and for five freeze/thaw cycles.

## Secondary outcomes: PSQI Scoring

### **Table S1.** Revised scoring of PSQI item 4 – hours of actual sleep.

| Original scoring |  | Revised scoring |  |
| --- | --- | --- | --- |
| > 7 hours | 0 | > 7 hours | 0 |
| 6-7 hours | 1 | >6 and ≤7 hours | 1 |
| 5-6 hours | 2 | ≥ 5 and ≤ 6 hours | 2 |
| < 5 hours | 3 | < 5 hours | 3 |

# SUPPLEMENTAL RESULTS

## **Results:** Analysis of JNJ-42165279

No participant in the PBO group had a detectable level of JNJ-42165279. Plasma concentrations of JNJ-42165279 in the FAAHi group declined from 178±114 (n=47) at week 4, to 129±89 (n=44) at week 8 and then further dropped to 86±88 (n=45) at week 12 (Figure 6a). However, many participants took the study drug the same day as the blood sample was drawn, especially at the first time at visit 4, explaining the decreasing trend over time. Plasma concentration levels should therefore not be interpreted as trough levels, but rather as an indicator of adherence. When testing for correlation between drug levels and change in clinician assessed PTSD symptoms (CAPS-5 severity scores) over the 12-week study period, we therefore only included participants in the FAAHi group who reported the last previous drug intake to 9 to 15 hours before the blood sample was taken (n=0 at week 4, n=25 at week 8, n=30 at week 12). We found no correlations between CAPS-5 scores and plasma concentrations of JNJ-42165279 at week 8 (r_s_[23] =.25, p=.24) nor at week 12 (r_s_[28]=.042, p=.83).

## **Results**: Subjective units of distress

The effect of treatment group on subjective units of distress (SUD) following therapy modules was assessed using a linear mixed effects model with module as a repeated within-subjects measure and individual identity as a random intercept. There was a significant interaction between treatment group and module for the second module (β = -13.5, SE = 6.794, *t*_333_ = -1.987, *p* = 0.047), though *post-hoc* comparisons revealed no significant difference between FAAHi and PBO for SUD after the second module (ΔFAAHi – PBO = 7.24, SE = 5.69, *t*_304_ = 1.273, *p* = 0.99). There was a significant main effect of module for the third (β = 26.5, SE = 5.1, *t*_338_ = 5.15, *p* > 0.001), fourth (β = 28.9, SE = 5.1, *t*_336_ = 5.68, *p* > 0.001), fifth (β = 17.8, SE = 5.4, *t*_337_ = 3.31, *p* = 0.001), sixth (β = 21.1, SE = 5.7, *t*_337_ = 3.72, *p* > 0.001), and seventh (β = 17.8, SE = 6.4, *t*_339_ = 2.79, *p* = 0.006) modules, but not the second (β = 8.2, SE = 4.9, *t*_331_ = 1.67, *p* = 0.10) or eighth (β = -2.2, SE = 6.2, *t*_338_ = -0.35, *p* = 0.73) modules. There was no significant main effect of treatment group (β = 6.3, SE = 5.5, *t*_295_ = 1.128, *p* = 0.26).


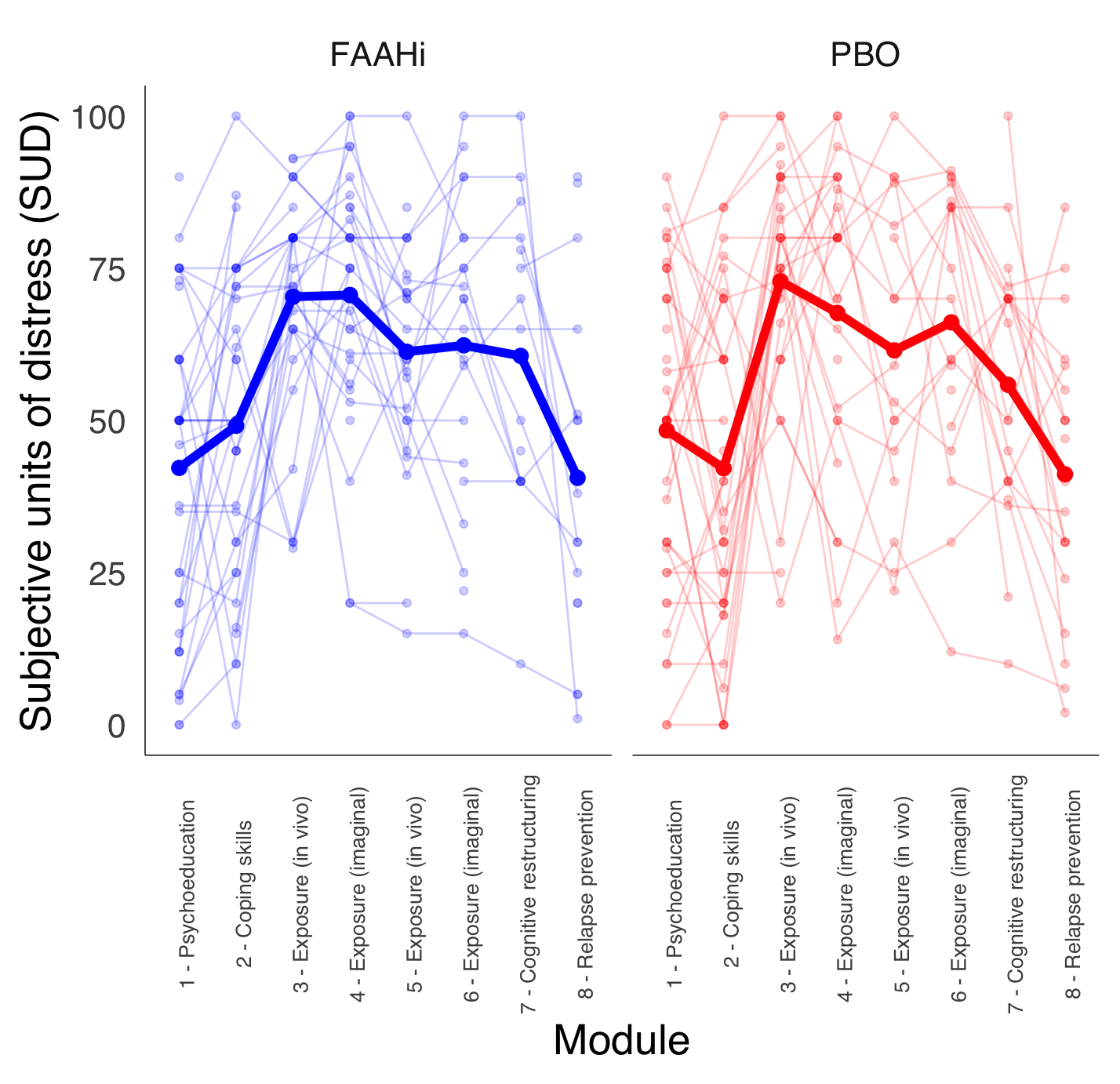


**Supplemental Figure S1. No significant difference in SUD between groups.** SUD were measured following each therapy module. Therapy included 8 modules completed over 8 weeks. Modules listed on the *x* axis include brief descriptions of what was covered in each respective module. Exposure occurred in modules 3-6.

## **Results:** PTSD symptom clusters

Separate linear mixed effects models with each sub-score as outcome were run using the R (version 4.3.1) package *lme4*. Subscale symptom severity was measured using the CAPS-5. Treatment group and week were included as fixed effects, and subject identity was included as a random intercept. There was a significant main effect of week for all four subscale models. There was no significant main effect of treatment group for any of the models. The interaction of treatment group and week was not significant for any of the models, though there was a trend towards significance for the arousal (*p* = 0.10) and avoidance (*p* = 0.09) subscale models.

**
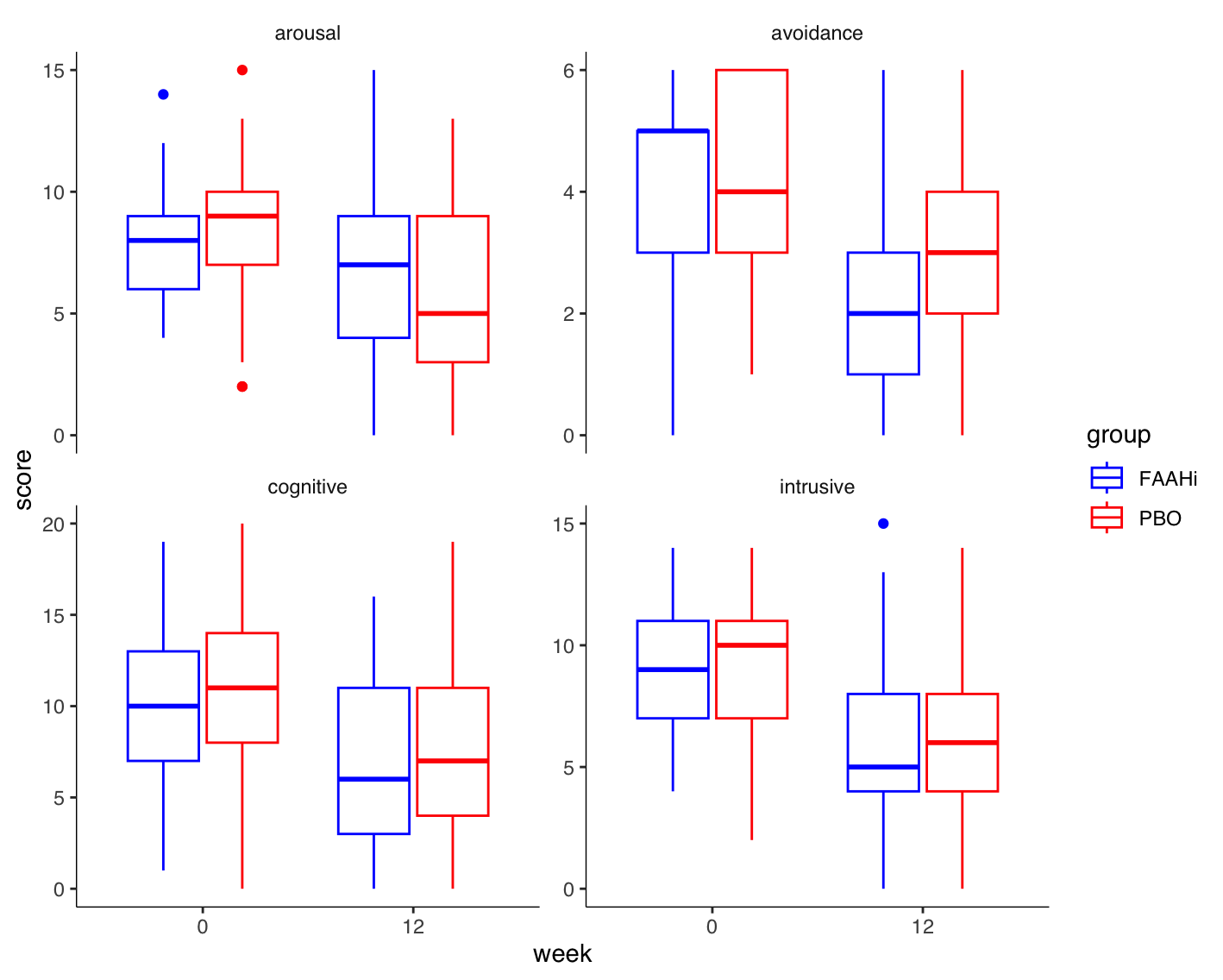
**

**Supplemental Figure 2: No significant effect of FAAH inhibition on any PTSD symptom subscale.** CAPS-5 subscales of Arousal, Avoidance, Cognitive, or Intrusive symptoms did not differ between FAAHi and PBO groups at baseline or week 12.

## **Table S2.** CAPS-5 subscales of PTSD symptom clusters

| **Model** | **Variable** | **Estimate** | **Std. Error** | ***t*-value** | ***p*-value** |
| --- | --- | --- | --- | --- | --- |
| Arousal | Intercept | 8.04 | 0.40 | *t*_164_ = 19.88 | < 0.001 *** |
|  | Group (PBO) | 0.31 | 0.58 | *t*_164_ = 0.53 | 0.59 |
|  | Week (12) | -1.32 | 0.49 | *t*_87_ = -2.72 | 0.008 ** |
|  | Group*Week | -1.18 | 0.70 | *t*_88_ = -1.68 | 0.10 |
| Avoidance | Intercept | 4.03 | 0.23 | *t*_167_ = 17.22 | < 0.001 *** |
|  | Group (PBO) | 0.06 | 0.34 | *t*_167_ = 0.19 | 0.85 |
|  | Week (12) | -1.94 | 0.29 | *t*_92_ = -6.78 | < 0.001 ** |
|  | Group*Week | 0.70 | 0.41 | *t*_93_ = -1.69 | 0.09 |
| Cognitive | Intercept | 9.96 | 0.63 | *t*_154_ = 15.89 | < 0.001 *** |
|  | Group (PBO) | 1.02 | 0.90 | *t*_154_ = 1.14 | 0.26 |
|  | Week (12) | -3.10 | 0.70 | *t*_87_ = -4.45 | < 0.001 ** |
|  | Group*Week | 0.08 | 1.00 | *t*_88_ = 0.08 | 0.94 |
| Intrusive | Intercept | 9.08 | 0.44 | *t*_159_ = 20.80 | < 0.001 *** |
|  | Group (PBO) | -0.06 | 0.62 | *t*_159_ = -0.09 | 0.93 |
|  | Week (12) | -3.19 | 0.50 | *t*_92_ = -6.40 | < 0.001 ** |
|  | Group*Week | 0.52 | 0.72 | *t*_93_ = 0.73 | 0.47 |

*** denotes significance at p > 0.001; ** denotes significance at p > 0.01.

## **Table S3.** Primary and secondary outcomes at each timepoint

|  |  | **Week 0** | **Week 4** | **Week 8** | **Week 12** | **Week 16** |
| --- | --- | --- | --- | --- | --- | --- |
| **PTSD severity score** (CAPS-5) | PBO | 32.4 (9.0) |  |  | 22.6 (10.7) | 20.0 (11.2) |
|  |  | 49 |  |  | 41 | 40 |
|  | FAAHi | 31.1 (7.1) |  |  | 21.4 (10.9) | 19.3 (12.4) |
|  |  | 51 |  |  | 45 | 45 |
| **Self-reported PTSD symptoms** (PCL-5) | PBO | 45.2 (13.6) | 37.8 (14.8) | 36.7 (13.2) | 30.6 (16.2) | 28.7 (15.1) |
|  |  | 49 | 48 | 43 | 41 | 34 |
|  | FAAHi | 43.4 (10.8) | 37.6 (13.9) | 36.6 (13.5) | 31.0 (18.0) | 27.9 (17.5) |
|  |  | 50 | 47 | 43 | 45 | 38 |
| **Anxiety symptoms** (CPRS-S-A) | PBO | 10.8 (4.4) |  |  | 9.0 (3.7) |  |
|  |  | 47 |  |  | 39 |  |
|  | FAAHi | 10.8 (3.9) |  |  | 9.0 (4.3) |  |
|  |  | 51 |  |  | 44 |  |
| **Depressive symptoms** (CPRS-S-A) | PBO | 10.6 (4.5) |  |  | 8.4 (4.6) |  |
|  |  | 45 |  |  | 37 |  |
|  | FAAHi | 10.3 (3.7) |  |  | 8.1 (4.8) |  |
|  |  | 51 |  |  | 43 |  |
| **Sleep quality** (PSQI) | PBO | 11.4 (3.9) | 11.3 (4.6) | 10.9 (3.9) | 10.0 (4.1) | 10.1 (4.0) |
|  |  | 43 | 43 | 40 | 32 | 27 |
|  | FAAHi | 10.6 (3.5) | 10.7 (3.6) | 10.4 (4.1) | 10.1 (4.0) | 9.7 (4.6) |
|  |  | 46 | 42 | 34 | 41 | 32 |
| Results presented as mean (SD) in the first row and then N below | | | | | | |

## **Table S4:** Effect of JNJ-42165279 on primary and secondary outcomes

| **Outcome** |  | **Estimate** | **Lower** | **Upper** | **p-value** |
| --- | --- | --- | --- | --- | --- |
| **PTSD severity score** (CAPS-5) | Baseline score | 31.77 | 30.17 | 33.37 | <.001 |
|  | Week 12 | -8.826 | -11.937 | -5.716 | <.001 |
|  | **Treatment effect** | **-1.063** | **-5.271** | **3.145** | **0.617** |
| **Self-reported PTSD symptoms** (PCL-5) | Baseline score | 44.31 | 41.879 | 46.741 | <.001 |
|  | Week 12 | -13.703 | -18.806 | -8.601 | <.001 |
|  | **Treatment effect** | **0.726** | **-6.15** | **7.601** | **0.834** |
| **Anxiety symptoms** (CPRS-S-A) | Baseline score | 10.813 | 9.993 | 11.632 | <.001 |
|  | Week 12 | -1.559 | -2.745 | -0.373 | 0.011 |
|  | **Treatment effect** | **-0.245** | **-1.769** | **1.279** | **0.75** |
| **Depressive symptoms** (CPRS-S-A) | Baseline score | 10.452 | 9.631 | 11.273 | <.001 |
|  | Week 12 | -2.04 | -3.461 | -0.619 | 0.005 |
|  | **Treatment effect** | **-0.214** | **-2.079** | **1.651** | **0.82** |
| **Sleep quality** (PSQI) | Baseline score | 11.005 | 10.239 | 11.771 | <.001 |
|  | Week 12 | -1.282 | -2.464 | -0.099 | 0.034 |
|  | **Treatment effect** | **0.767** | **-0.791** | **2.324** | **0.329** |

*Baseline scores:* baseline (week 0) scores for the whole study population (n=100) for each outcome. *Week 12:* score change in each outcome measure from week 0 to week 12. *Treatment effect*: the effect of the study drug on score change in each outcome measure between 0 and 12 weeks. *Lower* and *upper*: 95% CI for the estimated coefficient.

## **Table S5:** Treatment effect after adjusting for Baseline (week 0) scores

| **Outcome** | **Characteristic** | **Beta** | **95% CI** | **p-value** |
| --- | --- | --- | --- | --- |
| **PTSD severity score** (CAPS-5) | baseline | 0.61 | 0.32, 0.89 | <0.001 |
|  | FAAHi | -1.1 | -5.3, 3.2 | 0.6 |
| **Self-reported PTSD symptoms** (PCL-5) | baseline | 0.52 | 0.23, 0.81 | <0.001 |
|  | FAAHi | 0.68 | -6.3, 7.7 | 0.8 |
| **Anxiety symptoms** (CPRS-S-A) | baseline | 0.50 | 0.31, 0.70 | <0.001 |
|  | FAAHi | -0.22 | -1.8, 1.3 | 0.8 |
| **Depressive symptoms** (CPRS-S-A) | baseline | 0.57 | 0.33, 0.82 | <0.001 |
|  | FAAHi | -0.17 | -2.1, 1.8 | 0.9 |
| **Sleep quality** (PSQI) | baseline | 0.70 | 0.47, 0.94 | <0.001 |
|  | FAAHi | 0.71 | -0.91, 2.3 | 0.4 |

## **Table S6:** Treatment effect after adjusting for baseline (week 0) AEA

| **Outcome** | **Characteristic** | **Beta** | **95% CI** | **p-value** |
| --- | --- | --- | --- | --- |
| **PTSD severity score** (CAPS-5) | baseline AEA | -5.6 | -15, 3.9 | 0.3 |
|  | FAAHi | -7.6 | -20, 4.5 | 0.2 |
|  | baseline AEA*FAAHi | 7.8 | -6.8, 22 | 0.3 |

## **Table S7:** Adverse events

| **Table S7.** Adverse events. | | | | |
| --- | --- | --- | --- | --- |
|  | **Overall (**n=100) | **PBO (**n=49) | **FAAHi** (n=51) |  |
|  | **n (%)** | **n (%)** | **n (%)** | **p-value^1^** |
| Any AE | 84 (84%) | 42 (85.7%) | 42 (82.4%) | 0.65 |
| Any SAE | 0 | 2 (4.1%) | 0 | 0.24 |
| Any AE leading to withdrawal | 0 | 1 (2.0%) | 0 | 0.49 |
| **Adverse events, by preferred term^2^** | | | |  |
| Fatigue | 31 (31.0%) | 13 (26.5%) | 18 (35.3%) | 0.27 |
| Headache | 28 (28.0%) | 14 (28.6%) | 14 (27.5%) | 1.0 |
| Dizziness | 11 (11.0%) | 4 (8.2%) | 7 (13.7%) | 0.37 |
| Anxiety | 11 (11.0%) | 5 (10.2%) | 6 (11.8%) | 0.76 |
| Nasopharyngitis | 10 (10.0%) | 4 (8.2%) | 6 (11.8%) | 0.53 |
| Depressed mood | 8 (8.0%) | 3 (6.1%) | 5 (9.8%) | 0.49 |
| Nightmare | 8 (8.0%) | 3 (6.1%) | 5 (9.8%) | 0.49 |
| Insomnia | 9 (9.0%) | 5 (10.2%) | 4 (7.8%) | 1.0 |
| Dry mouth | 8 (8.0%) | 4 (8.2%) | 4 (7.8%) | 1.0 |
| Abdominal pain | 6 (6.0%) | 2 (4.1%) | 4 (7.8%) | 0.44 |
| Unintentional weight gain | 6 (6.0%) | 2 (4.1%) | 4 (7.8%) | 0.44 |
| Constipation | 5 (5.0%) | 1 (2.0%) | 4 (7.8%) | 0.2 |
| Decreased appetite | 6 (6.0%) | 3 (6.1%) | 3 (5.9%) | 1.0 |
| Influenza | 4 (4.0%) | 1 (2.0%) | 3 (5.9%) | 0.36 |
| Disturbance of attention | 3 (3.0%) | 0 (0.0%) | 3 (5.9%) | 0.11 |
| Paraesthesia | 6 (6.0%) | 4 (8.2%) | 2 (3.9%) | 0.69 |
| Covid-19 | 5 (5.0%) | 3 (6.1%) | 2 (3.9%) | 1.0 |
| Menstruation irregular | 5 (5.0%) | 3 (6.1%) | 2 (3.9%) | 1.0 |
| Nausea | 7 (7.0%) | 6 (12.2%) | 1 (2.0%) | 0.12 |
| Sleep disorder | 4 (4.0%) | 3 (6.1%) | 1 (2.0%) | 0.62 |
| Memory impairment | 4 (4.0%) | 4 (8.2%) | 0 (0.0%) | 0.12 |
| Blood potassium decreased | 3 (3.0%) | 3 (6.1%) | 0 (0.0%) | 0.25 |
| Libido decreased | 3 (3.0%) | 3 (6.1%) | 0 (0.0%) | 0.25 |
| ^1^ Chi-square (Pearson or Fischer’s exact test).  ^2^ Occurring in ≥5% of participants in any treatment arm. | | | | |

## **Table S8:** All treatment emergent adverse events by System organ class and preferred term.

| **System organ class** | **Preferred term** | **Overall** | | **PBO** | | **FAAHi** | |
| --- | --- | --- | --- | --- | --- | --- | --- |
|  |  | **n** | **%** | **n** | **%** | **n** | **%** |
| **Blood and lymphatic system disorders** | Anaemia | 2 | 2.0 | 0 | 0.0 | 2 | 3.9 |
|  | Increased tendency to bruise | 1 | 1.0 | 0 | 0.0 | 1 | 2.0 |
| **Cardiac disorders** | Palpitation | 4 | 4.0 | 2 | 4.1 | 2 | 3.9 |
| **Ear and labyrinth disorders** | Ear discomfort | 1 | 1.0 | 0 | 0.0 | 1 | 2.0 |
|  | Hyperacusis | 2 | 2.0 | 1 | 2.0 | 1 | 2.0 |
|  | Tinnitus | 2 | 2.0 | 1 | 2.0 | 1 | 2.0 |
| **Eye disorders** | Eye pain | 1 | 1.0 | 0 | 0.0 | 1 | 2.0 |
|  | Hordeolum | 1 | 1.0 | 0 | 0.0 | 1 | 2.0 |
|  | Vision blurred | 3 | 3.0 | 2 | 4.1 | 1 | 2.0 |
|  | Vision decreased | 1 | 1.0 | 1 | 2.0 | 0 | 0.0 |
| **Gastrointestinal disorders** | Abdominal discomfort | 2 | 2.0 | 1 | 2.0 | 1 | 2.0 |
|  | Abdominal pain | 6 | 6.0 | 2 | 4.1 | 4 | 7.8 |
|  | Ageusia | 1 | 1.0 | 1 | 2.0 | 0 | 0.0 |
|  | Anal pruritus | 1 | 1.0 | 0 | 0.0 | 1 | 2.0 |
|  | Bowel movement irregularity | 1 | 1.0 | 0 | 0.0 | 1 | 2.0 |
|  | Constipation | 5 | 5.0 | 1 | 2.0 | 4 | 7.8 |
|  | Diarrhoea | 3 | 3.0 | 1 | 2.0 | 2 | 3.9 |
|  | Dry mouth | 8 | 8.0 | 4 | 8.2 | 4 | 7.8 |
|  | Dyspepsia | 3 | 3.0 | 2 | 4.1 | 1 | 2.0 |
|  | Dysphagia | 1 | 1.0 | 1 | 2.0 | 0 | 0.0 |
|  | Faeces discoloration | 1 | 1.0 | 1 | 2.0 | 0 | 0.0 |
|  | Flatulence | 1 | 1.0 | 0 | 0.0 | 1 | 2.0 |
|  | Gastritis | 1 | 1.0 | 0 | 0.0 | 1 | 2.0 |
|  | Glossodynia | 1 | 1.0 | 0 | 0.0 | 1 | 2.0 |
|  | Mouth ulceration | 1 | 1.0 | 0 | 0.0 | 1 | 2.0 |
|  | Nausea | 7 | 7.0 | 6 | 12.2 | 1 | 2.0 |
| **General disorders** | Abnormal (unintentional) weight gain | 6 | 6.0 | 2 | 4.1 | 4 | 7.8 |
|  | Cold sweat | 1 | 1.0 | 1 | 2.0 | 0 | 0.0 |
|  | Decreased appetite | 6 | 6.0 | 3 | 6.1 | 3 | 5.9 |
|  | Fatigue | 31 | 31.0 | 13 | 26.5 | 18 | 35.3 |
|  | Feeling cold | 2 | 2.0 | 1 | 2.0 | 1 | 2.0 |
|  | Hunger | 3 | 3.0 | 2 | 4.1 | 1 | 2.0 |
|  | Hyperhidrosis | 1 | 1.0 | 1 | 2.0 | 0 | 0.0 |
|  | Increased appetite | 3 | 3.0 | 1 | 2.0 | 2 | 3.9 |
|  | Irritability | 1 | 1.0 | 0 | 0.0 | 1 | 2.0 |
|  | Night sweats | 1 | 1.0 | 0 | 0.0 | 1 | 2.0 |
|  | Oedema | 2 | 2.0 | 1 | 2.0 | 1 | 2.0 |
|  | Pain | 2 | 2.0 | 1 | 2.0 | 1 | 2.0 |
|  | Pyrexia | 3 | 3.0 | 2 | 4.1 | 1 | 2.0 |
|  | Thirst | 3 | 3.0 | 1 | 2.0 | 2 | 3.9 |
| **Hepatobiliary disorders** | Liver pain | 1 | 1.0 | 1 | 2.0 | 0 | 0.0 |
| **Infections and infestations** | Covid-19 | 5 | 5.0 | 3 | 6.1 | 2 | 3.9 |
|  | Diverticulitis | 1 | 1.0 | 1 | 2.0 | 0 | 0.0 |
|  | Fungal infection | 1 | 1.0 | 1 | 2.0 | 0 | 0.0 |
|  | Gastroenteritis viral | 2 | 2.0 | 1 | 2.0 | 1 | 2.0 |
|  | Influenza | 4 | 4.0 | 1 | 2.0 | 3 | 5.9 |
|  | Nail infection | 1 | 1.0 | 1 | 2.0 | 0 | 0.0 |
|  | Nasopharyngitis | 10 | 10.0 | 4 | 8.2 | 6 | 11.8 |
|  | Peritonsillar abscess | 1 | 1.0 | 1 | 2.0 | 0 | 0.0 |
|  | Pyelonephritis | 1 | 1.0 | 0 | 0.0 | 1 | 2.0 |
|  | Tonsillitis | 1 | 1.0 | 1 | 2.0 | 0 | 0.0 |
|  | Urinary tract infection | 3 | 3.0 | 2 | 4.1 | 1 | 2.0 |
|  | Vulvovaginal fungal infection | 1 | 1.0 | 1 | 2.0 | 0 | 0.0 |
| **Injury, poisoning and procedural complications** | Contusion | 1 | 1.0 | 0 | 0.0 | 1 | 2.0 |
|  | Fall | 3 | 3.0 | 1 | 2.0 | 2 | 3.9 |
|  | Food poisoning | 1 | 1.0 | 1 | 2.0 | 0 | 0.0 |
|  | Injury | 1 | 1.0 | 0 | 0.0 | 1 | 2.0 |
|  | Poisoning | 1 | 1.0 | 1 | 2.0 | 0 | 0.0 |
| **Investigations** | Alanine transferase increased | 2 | 2.0 | 1 | 2.0 | 1 | 2.0 |
|  | Aspartate aminotransferase increased | 1 | 1.0 | 1 | 2.0 | 0 | 0.0 |
|  | Blood albumin increased | 1 | 1.0 | 1 | 2.0 | 0 | 0.0 |
|  | Blood alkaline phosphate increased | 1 | 1.0 | 0 | 0.0 | 1 | 2.0 |
|  | Blood calcium decreased | 2 | 2.0 | 1 | 2.0 | 1 | 2.0 |
|  | Blood creatinine increased | 1 | 1.0 | 0 | 0.0 | 1 | 2.0 |
|  | Blood potassium decreased | 3 | 3.0 | 3 | 6.1 | 0 | 0.0 |
|  | Blood pressure decreased | 1 | 1.0 | 0 | 0.0 | 1 | 2.0 |
|  | CRP increased | 1 | 1.0 | 1 | 2.0 | 0 | 0.0 |
|  | Haemoglobin decreased | 3 | 3.0 | 1 | 2.0 | 2 | 3.9 |
|  | Phosphatidylethanol increased | 1 | 1.0 | 1 | 2.0 | 0 | 0.0 |
|  | Platelet count increased | 1 | 1.0 | 1 | 2.0 | 0 | 0.0 |
| **Metabolism and nutrition disorders** | Salt craving | 1 | 1.0 | 1 | 2.0 | 0 | 0.0 |
| **Musculoskeletal and connective tissue disorders** | Arthralgia | 2 | 2.0 | 2 | 4.1 | 0 | 0.0 |
|  | Back pain | 2 | 2.0 | 1 | 2.0 | 1 | 2.0 |
|  | Joint stiffness | 1 | 1.0 | 0 | 0.0 | 1 | 2.0 |
|  | Muscle spasm | 2 | 2.0 | 1 | 2.0 | 1 | 2.0 |
|  | Pain in extremity | 1 | 1.0 | 0 | 0.0 | 1 | 2.0 |
| **Nervous system disorders** | Burning sensation | 1 | 1.0 | 0 | 0.0 | 1 | 2.0 |
|  | Dizziness | 11 | 11.0 | 4 | 8.2 | 7 | 13.7 |
|  | Headache | 28 | 28.0 | 14 | 28.6 | 14 | 27.5 |
|  | Hypoaesthesia | 4 | 4.0 | 2 | 4.1 | 2 | 3.9 |
|  | Paraesthesia | 6 | 6.0 | 4 | 8.2 | 2 | 3.9 |
|  | Parosmia | 1 | 1.0 | 1 | 2.0 | 0 | 0.0 |
|  | Presyncope | 2 | 2.0 | 0 | 0.0 | 2 | 3.9 |
|  | Skin burning sensation | 1 | 1.0 | 1 | 2.0 | 0 | 0.0 |
| **Psychiatric disorders** | Abnormal dreams | 3 | 3.0 | 2 | 4.1 | 1 | 2.0 |
|  | Affect lability | 1 | 1.0 | 1 | 2.0 | 0 | 0.0 |
|  | Anger | 1 | 1.0 | 1 | 2.0 | 0 | 0.0 |
|  | Anxiety | 11 | 11.0 | 5 | 10.2 | 6 | 11.8 |
|  | Apathy | 1 | 1.0 | 1 | 2.0 | 0 | 0.0 |
|  | Blunted affect | 1 | 1.0 | 1 | 2.0 | 0 | 0.0 |
|  | Bradyphrenia | 2 | 2.0 | 2 | 4.1 | 0 | 0.0 |
|  | Depressed mood | 8 | 8.0 | 3 | 6.1 | 5 | 9.8 |
|  | Disturbance of attention | 3 | 3.0 | 0 | 0.0 | 3 | 5.9 |
|  | Emotional poverty | 1 | 1.0 | 1 | 2.0 | 0 | 0.0 |
|  | Hallucination, auditory | 1 | 1.0 | 1 | 2.0 | 0 | 0.0 |
|  | Hypervigilans | 1 | 1.0 | 1 | 2.0 | 0 | 0.0 |
|  | Increased PTSD symptoms | 1 | 1.0 | 0 | 0.0 | 1 | 2.0 |
|  | Insomnia | 9 | 9.0 | 5 | 10.2 | 4 | 7.8 |
|  | Memory impairment | 4 | 4.0 | 4 | 8.2 | 0 | 0.0 |
|  | Mood swings | 1 | 1.0 | 0 | 0.0 | 1 | 2.0 |
|  | Nightmare | 8 | 8.0 | 3 | 6.1 | 5 | 9.8 |
|  | Sleep disorder | 4 | 4.0 | 3 | 6.1 | 1 | 2.0 |
|  | Suicidal ideation | 1 | 1.0 | 0 | 0.0 | 1 | 2.0 |
|  | Thinking abnormal | 1 | 1.0 | 1 | 2.0 | 0 | 0.0 |
| **Renal and urinary tract disorders** | Dysuria | 2 | 2.0 | 1 | 2.0 | 1 | 2.0 |
|  | Haematuria | 1 | 1.0 | 1 | 2.0 | 0 | 0.0 |
|  | Pollakiuria | 2 | 2.0 | 1 | 2.0 | 1 | 2.0 |
| **Reproductive system and breast disorders** | Breast pain | 2 | 2.0 | 2 | 4.1 | 0 | 0.0 |
|  | Libido decreased | 3 | 3.0 | 3 | 6.1 | 0 | 0.0 |
|  | Menstruation irregular | 5 | 5.0 | 3 | 6.1 | 2 | 3.9 |
|  | Vulvovaginal dryness | 1 | 1.0 | 0 | 0.0 | 1 | 2.0 |
| **Respiratory, thoracic and mediastinal disorders** | Cough | 1 | 1.0 | 1 | 2.0 | 0 | 0.0 |
|  | Dyspnoea | 1 | 1.0 | 0 | 0.0 | 1 | 2.0 |
|  | Hoarsness | 2 | 2.0 | 1 | 2.0 | 1 | 2.0 |
|  | Nasal congestion | 2 | 2.0 | 1 | 2.0 | 1 | 2.0 |
|  | Oropharyngeal pain | 2 | 2.0 | 0 | 0.0 | 2 | 3.9 |
|  | Yawning | 1 | 1.0 | 1 | 2.0 | 0 | 0.0 |
| **Skin and subcutaneous tissue disorders** | Acne | 1 | 1.0 | 1 | 2.0 | 0 | 0.0 |
|  | Alopecia | 1 | 1.0 | 1 | 2.0 | 0 | 0.0 |
|  | Dry skin | 3 | 3.0 | 1 | 2.0 | 2 | 3.9 |
|  | Ingrowing nail | 1 | 1.0 | 1 | 2.0 | 0 | 0.0 |
|  | Onychoclasis | 1 | 1.0 | 0 | 0.0 | 1 | 2.0 |
|  | Pain of skin | 1 | 1.0 | 1 | 2.0 | 0 | 0.0 |
|  | Petechiae | 1 | 1.0 | 0 | 0.0 | 1 | 2.0 |
|  | Piloerection | 1 | 1.0 | 1 | 2.0 | 0 | 0.0 |
|  | Pruritus | 1 | 1.0 | 1 | 2.0 | 0 | 0.0 |
|  | Rash | 1 | 1.0 | 1 | 2.0 | 0 | 0.0 |
|  | Sensitive skin | 1 | 1.0 | 1 | 2.0 | 0 | 0.0 |
|  | Skin odor abnormal | 1 | 1.0 | 1 | 2.0 | 0 | 0.0 |
|  | Urticaria | 1 | 1.0 | 1 | 2.0 | 0 | 0.0 |
| **Surgical and medical procedures** | Hospitalisation | 1 | 1.0 | 1 | 2.0 | 0 | 0.0 |
